# Low-Grade Mucoepidermoid Carcinoma of the Parotid in a Pediatric Survivor of Hodgkin’s Lymphoma

**DOI:** 10.7759/cureus.94346

**Published:** 2025-10-11

**Authors:** Sabeen Wazir, Kyra Salinkas, Berony Geneste, Jodie Shao, John M Lipka

**Affiliations:** 1 Medicine, Edward Via College of Osteopathic Medicine, Monroe, USA; 2 Medicine, Texas Tech University Health Sciences Center El Paso, Paul L. Foster School of Medicine, El Paso, USA; 3 Surgery, Edward Via College of Osteopathic Medicine, Monroe, USA

**Keywords:** hodgkin lymphona, long-term outcome, mucoepidermoid carcinoma (mec), pediatric hematology-oncology, salivary gland cancer, total parotidectomy

## Abstract

Mucoepidermoid carcinoma (MEC) is an uncommon malignancy of the salivary glands in the pediatric population. MEC typically occurs as a secondary malignancy in patients who have undergone radiation therapy for cancer. The occurrence of MEC in patients who have undergone chemotherapy without prior radiotherapy is extremely rare. A 17-year-old female patient with a history of Hodgkin’s lymphoma and anemia presented to the clinic with a painless hard lump between her mastoid and mandibular process. The patient's history revealed that at 11 years of age, she was diagnosed with stage IIIB Hodgkin’s disease and received the ABVE-PC chemotherapy regimen. This regimen includes doxorubicin (Adriamycin), bleomycin, vincristine, etoposide, prednisone, and cyclophosphamide. The lack of bulk disease and complete response after two cycles of chemotherapy excluded her from receiving involved field radiation therapy (IFRT). Physical examination of the neck revealed a ~1.5 cm firm, non-tender mass near the left parotid. Fine needle aspiration biopsy results revealed spindle-shaped cells and unusual mitoses, and a superficial parotidectomy was recommended. Genetic testing for major oncogenes and tumor suppressor gene mutations was conducted. Results were negative for any significant genetic factors. Alkylating chemotherapy agents and immunosuppression increase the risk of secondary malignancies, although they have not been directly linked to MEC. This case suggests a potential association between chemotherapy treatment and MEC. Furthermore, it raises the question of whether increased vigilance and long-term follow-up are necessary for this vulnerable population of childhood cancer survivors who have not received radiation therapy.

## Introduction

Head and neck cancers account for 4% of all cancers in the U.S, and 1% of these cancers are salivary gland cancers (SGC) [1]. Mucoepidermoid carcinoma (MEC) is an SGC that accounts for 35% of all SGCs [2]. Pediatric cases comprise only 5% of MEC diagnoses, making it an exceedingly rare malignancy in children [1,2]. While radiation therapy is a well-documented risk factor for secondary MEC, the potential role of chemotherapy in the development of MEC remains unclear [3]. 

The prognosis of pediatric MEC patients largely depends on the histological grade and clinical presentation of the cancer [4]. Histologically, MEC is characterized by the presence of squamous cells, goblet mucin-secreting cells, and intermediate type cells, and is classified into three grades: low, intermediate, and high [5]. Standard treatment typically involves surgical removal of the primary tumor, often followed by radiation therapy, particularly for high-grade tumors or cases with incomplete resection [4]. 

Here, we present a rare case of a pediatric patient diagnosed with MEC of the parotid gland, with a past medical history of Stage IIIB Hodgkin lymphoma treated without radiation therapy. We believe that the patient’s chemotherapy may be a contributing factor to her disease and hope to raise awareness about the possibility of chemotherapy linked to secondary SGCs in pediatric lymphoma and leukemia patients.

## Case presentation

A 17-year-old female patient, with a history of nodular sclerosing Hodgkin Lymphoma and anemia, presented to the clinic with a painless hard lump between her mastoid and mandibular process. The patient reported that the lump was first noticed nine months prior and had enlarged very slowly.

At the age of 11, the patient had been diagnosed with stage IIIB Hodgkin disease. The cancer had been treated with the ABVE-PC regimen, which includes doxorubicin (Adriamycin), bleomycin, vincristine, etoposide, prednisone, and cyclophosphamide. A lack of bulk disease and a complete response after two cycles of chemotherapy excluded her from receiving involved-field radiation therapy (IFRT). The patient had an extensive surgical history, including tonsillectomy and adenoidectomy (age five), abdominal laparoscopy (age 11), abdominal laparotomy (age 11), bone marrow biopsy (age 11), and port-catheter placement surgery (age 11) and its removal (age 12). 

**Figure 1:**
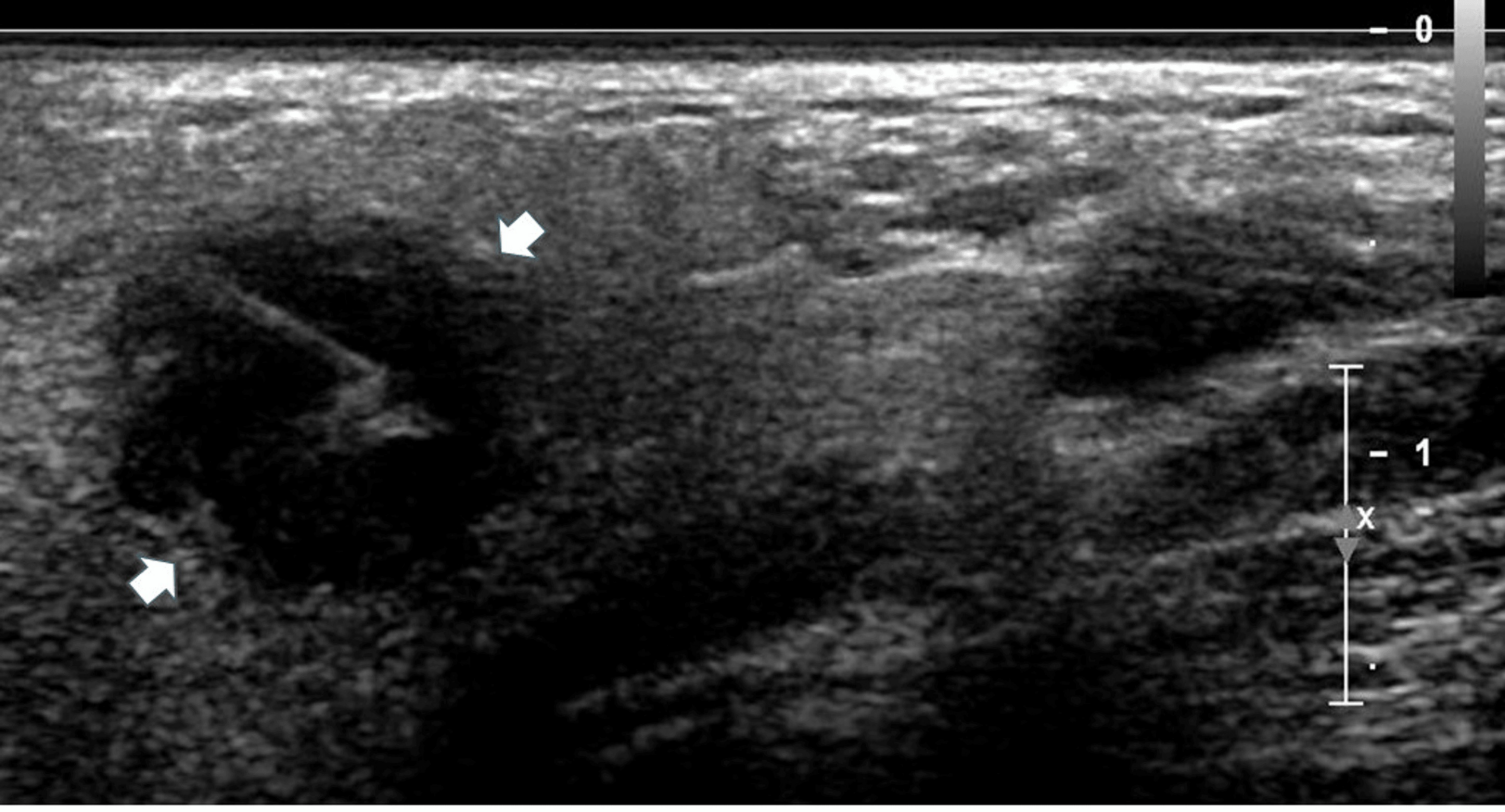
Fine needle aspiration ultrasound image of the mass (indicated by the white arrows) Needle is apparent in the mass. Tissue was obtained for histological examination.

Upon presentation, the patient appeared well-nourished and well-developed, with no indications of distress. Furthermore, she denied all other symptoms at that time. Physical examination of the neck revealed a ~1.5 cm firm non-tender mass by the tail of the left parotid. Laboratory findings were unremarkable. A fine needle aspiration (FNA) biopsy under ultrasound (US) guidance revealed low-grade epithelial proliferation with marked fibrosis and acute and chronic inflammation, raising suspicion for an epithelial neoplasm (Figure 1). 

The FNA biopsy results were ultimately inconclusive. However, a negative CD15 and CD30 staining indicated no reoccurrence of Hodgkin disease. After considering the patient’s past medical history and the inability to rule the pathology as benign, a superficial parotidectomy was recommended (Table 1). 

**Table 1:** Histological staining results of the fine needle aspirate from the left parotid gland CD: Cluster of Differentiation; PAX5: Paired Box 5.

Overall finding	Epithelial proliferation, suspicious for low-grade epithelial neoplasm
CD45 stain	Inflammatory infiltrates
CD20 and PAX5 stain	Infiltrating B cells
CD15 stain	Negative
CD30 stain	Negative
Differential diagnosis	Sclerosing polycystic adenosis, Cystadenoma, Sclerosing mucoepidermoid carcinoma, and reactive/inflammatory conditions

During the procedure, histologic findings revealed a secondary malignancy of low-grade MEC, and a total parotidectomy was performed.

**Figure 2:**
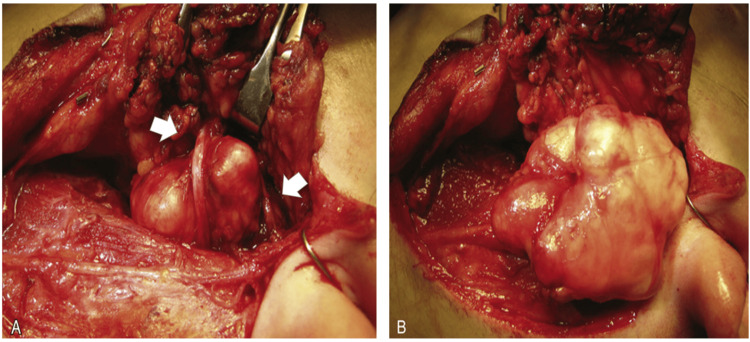
Gross total parotidectomy with diligence to the facial nerve Arrows indicate the tumor [6]. Facial nerve was apparent on the tumor, and the real tumor dimensions were 3.3 x 2.9 x 1.3 cm.

The total parotidectomy was carefully and meticulously performed to minimize both facial nerve paralysis and cosmetic differences. A modified Blair incision was planned, and care was taken in the area of the mass to not violate the area around it. The tragal pointer was identified, and the main trunk of the facial nerve was identified in its most common anatomical position. Both the upper and lower branches were identified and protected. The lower division of the nerve was traced distally, and several branches were encountered and dissected. Parotid tissue superior to the nerve was divided. Once anterior to the mass, it was excised completely, along with a generous cuff of normal parotid tissue (Figure 2).

The facial nerve was stimulated at the end of the surgery, indicating nerve function was within normal limits. The wound bed was irrigated with copious normal saline, and a Valsalva maneuver was performed. Hemostasis was excellent. A Jackson-Pratt drain (Teleflex, Wayne, Pennsylvania, US) was left in place. The wound was closed in a layered fashion with 4-0 vicryl and 5-0 fast gut (both by Ethicon, Inc., Ohio, US). Bacitracin was applied, as well as a head wrap dressing. The patient was then returned to anesthesia, was extubated in a routine fashion and transported to the recovery room in a good condition.

The patient recovered well; however, during her one-week post-operative follow-up, she reported pain upon beginning a meal, which was subsequently diagnosed as first bite syndrome (FBS). This complication began shortly i.e. two days after completion of the surgery. Due to the second malignancy, genetic karyotyping testing for major tumor suppressor and oncogenes was conducted. Results were negative for any significant genetic factors. Five years later, the patient was diagnosed with a benign neoplasm of the shoulder.

## Discussion

MEC in a pediatric patient as a secondary malignancy is extremely rare. Head and neck radiation therapy increases the risk for SGC as a secondary malignancy by 17-fold; without radiation, it is incredibly rare to develop MEC as a secondary malignancy [3]. Currently, there are no studies linking a particular chemotherapy reagent to MECs. However, it is not unheard of for patients with chemotherapy-only regimens to develop MEC as a secondary malignancy. Alkylating chemotherapy agents and topoisomerase II inhibitors such as cyclophosphamide and etoposide, respectively, increase the risk for secondary malignancies through their genotoxic mechanisms, which induce DNA damage and promote mutagenesis in hematopoietic and other susceptible cells [7,8].

We conducted a PubMed search using the terms “Mucoepidermoid Carcinoma” AND “Chemotherapy” AND “Secondary Malignancy.” These terms yielded two matched papers and 108 similar papers. Abstracts were reviewed to exclude articles that discussed cases of MEC induced by radiation therapy. Full-text articles were chosen for data extraction. Studies not written in English were excluded. Only published case reports, case series, original articles, review articles, and meta-analyses were included. After applying the inclusion and exclusion criteria, a total of six studies and 13 patient cases were selected. 

Upon observation, it was noted that all the patients with a secondary diagnosis of MEC had standard therapy regimens that included cyclophosphamide, strengthening the case that alkylating chemotherapy agents may play a role in developing secondary MEC. After factoring in the results of the literature search and the present patient’s case, the average years between primary and secondary cancer diagnosis was 5.8 years (Table 2).

**Table 2:** Results from a PubMed search using the terms “mucoepidermoid carcinoma”, “chemotherapy” and “secondary malignancy” ALL: Acute Lymphoblastic Leukemia, AML: Acute Myeloid Leukemia, TBI: total body irradiation, C*: chemotherapy.

Type of primary cancer	Years between primary and secondary malignancy	Alkylating agents	Etoposide (Y/N)	Notes	Reference
ALL	10	C*			[9]
ALL	4	Cyclophosphamide	N		[10]
ALL	13	C*		TBI	[2]
ALL	6.5	C*		TBI	[2]
ALL	3	C*			[2]
AML	7	Cyclophosphamide		TBI	[11]
AML	7	Busulfan Cyclophosphamide	Y		[8]
B-cell lymphoma	1	C*			[2]
Ewing sarcoma	11	Cyclophosphamide	N		[7]
Non-Hodgkin’s lymphoma	4	C*			[2]
Neuroblastoma	1	Cyclophosphamide	Y		[12]
Osteosarcoma	2.25	Cyclophosphamide	N		[7]
Rhabdomyosarcoma	6	C*			[2]

This time frame demonstrated that more care and attention may be needed in the long-term management of childhood cancer survivors.

Treatment of MEC of the left parotid included a total parotidectomy, a rare surgery to conduct in pediatric patients. Total parotidectomies require utmost care due to the increased risk of facial nerve damage, causing permanent facial paralysis with consequential psychosocial effects [13,14]. Our patient reported experiencing FBS, a common side effect of parotidectomies. This syndrome causes patients to experience a severe spasm or cramping upon the first few bites of a meal within the parotid region [15]. This patient's FBS, unfortunately, remains permanent. 

**Figure 3:**
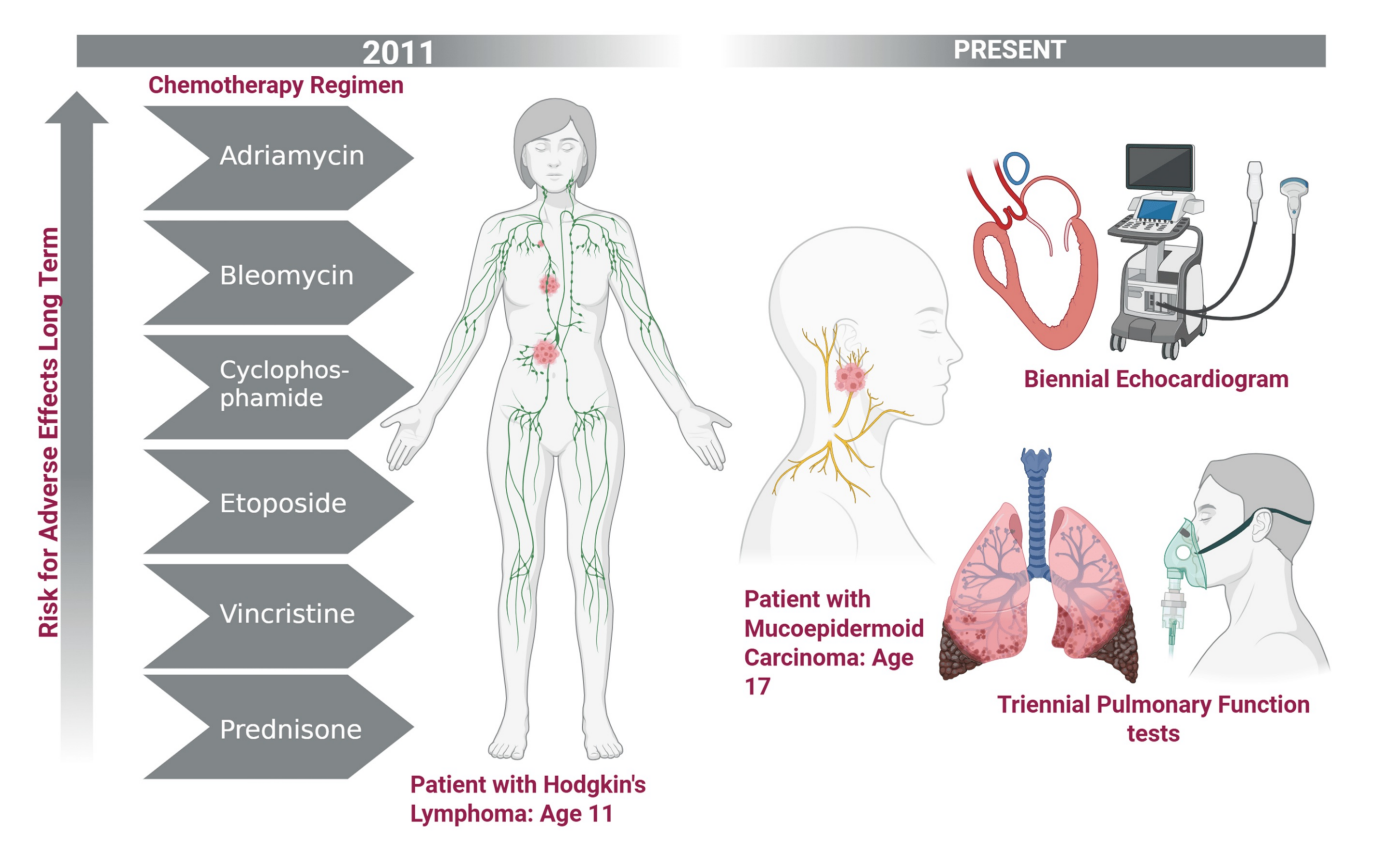
Plan for the long-term surveillance of chemotherapy-induced effects Created in BioRender. Wazir, S. (2025) https://BioRender.com/pcmn9me. Due to the patient's intensive chemotherapy, the patient will remain on triennial pulmonary function tests and biennial echocardiograms for the rest of their life.

Prior to the patient’s diagnosis, she had received extensive chemotherapy for which the long-term side effects must be evaluated through triennial pulmonary function tests, biennial echocardiograms, and annual complete blood counts (CBCs) for her lifetime (Figure 3).

Currently, the patient continues the previous after-cancer care; however, we recommend annual physical exams that pay additional attention to the neck and monthly self-breast exams. 

## Conclusions

MEC is rare in the pediatric population, especially as a secondary malignancy in patients without a history of radiation therapy. This patient’s clinical course underscores the importance of vigilant long-term follow-up in childhood cancer survivors, as they are at increased risk for developing secondary malignancies. Alkylating chemotherapy agents and immunosuppression increase the risk of secondary malignancies, though they have not been directly linked to MEC. This case suggests a potential association between chemotherapy treatment and the subsequent development of MEC, a rare but serious malignancy. This case further raises the question of whether more emphasis should be placed on early detection and intervention for this vulnerable population.
